# The Interplay between Intracellular Iron Homeostasis and Neuroinflammation in Neurodegenerative Diseases

**DOI:** 10.3390/antiox12040918

**Published:** 2023-04-12

**Authors:** Jaewang Lee, Dong-Hoon Hyun

**Affiliations:** Department of Life Science, Ewha Womans University, Seoul 03760, Republic of Korea

**Keywords:** intracellular iron homeostasis, neuroinflammation, neurodegenerative diseases, Nrf2, NF-κB, ferroptosis, 4-HNE

## Abstract

Iron is essential for life. Many enzymes require iron for appropriate function. However, dysregulation of intracellular iron homeostasis produces excessive reactive oxygen species (ROS) via the Fenton reaction and causes devastating effects on cells, leading to ferroptosis, an iron-dependent cell death. In order to protect against harmful effects, the intracellular system regulates cellular iron levels through iron regulatory mechanisms, including hepcidin–ferroportin, divalent metal transporter 1 (DMT1)–transferrin, and ferritin–nuclear receptor coactivator 4 (NCOA4). During iron deficiency, DMT1–transferrin and ferritin–NCOA4 systems increase intracellular iron levels via endosomes and ferritinophagy, respectively. In contrast, repleting extracellular iron promotes cellular iron absorption through the hepcidin–ferroportin axis. These processes are regulated by the iron-regulatory protein (IRP)/iron-responsive element (IRE) system and nuclear factor erythroid 2-related factor 2 (Nrf2). Meanwhile, excessive ROS also promotes neuroinflammation by activating the nuclear factor kappa-light-chain-enhancer of activated B cells (NF-κB). NF-κB forms inflammasomes, inhibits silent information regulator 2-related enzyme 1 (SIRT1), and induces pro-inflammatory cytokines (IL-6, TNF-α, and IL-1β). Furthermore, 4-hydroxy-2,3-*trans*-nonenal (4-HNE), the end-product of ferroptosis, promotes the inflammatory response by producing amyloid-beta (Aβ) fibrils and neurofibrillary tangles in Alzheimer’s disease, and alpha-synuclein aggregation in Parkinson’s disease. This interplay shows that intracellular iron homeostasis is vital to maintain inflammatory homeostasis. Here, we review the role of iron homeostasis in inflammation based on recent findings.

## 1. Introduction

Iron is a mineral nutrient essential for the survival of living organisms. It is a cofactor of many vital enzymes and has a crucial role as a heme component in transferring molecular oxygen to cells. Iron is known as the most abundant transition metal in the brain. However, iron does not exist in the brain at birth [1]. Instead, iron levels are drastically increased during adolescence and then maintained at constant levels [2]. Excessive iron can increase the labile iron pool (LIP), raising the levels of intracellular reactive oxygen species (ROS) [3,4,5,6], and iron depletion can promote the dysfunction of iron-dependent enzymes. Disruption of iron regulation is known to be involved in the pathogenesis of various neurodegenerative disorders [7,8,9,10]. Most of the total brain iron exists in the glial cells, such as astrocytes, oligodendrocytes, and microglia, rather than in the neurons [11] and is bound to ferritin, an iron storage protein [12]. Consequently, neurons are more vulnerable than glial cells to alterations in the iron balance.

Iron usually exists in two forms in the body: ferrous iron (Fe^2+^) and ferric iron (Fe^3+^) [13]. Fe^3+^ binds to transferrin (Tf), a bilobal protein, and forms the diferric Tf (Fe_2_Tf) complex, which circulates in the body [14]. In enterocytes, duodenal cytochrome B (DcytB) reduces Fe^3+^ of Fe_2_Tf to Fe^2+^, and divalent metal transporter 1 (DMT1) imports Fe^2+^ into the cells [15]. Transferrin receptor (TfR), one of the iron transporters, can also import Fe^2+^ into cells by forming vesicles, and then iron is stored complexed with ferritin, which is composed of ferritin heavy chain 1 (FTH1) and ferritin light chain (FTL) [16,17,18].

When more cellular iron is required, the cellular iron-regulatory protein (IRP)/iron-responsive element (IRE) system facilitates TfR expression, and stored iron (ferritin) is released through nuclear receptor coactivator 4 (NCOA4) activation [19,20]. By contrast, iron depletion increases iron storage and ferroportin 1 (FPN1) expression to reduce labile iron. This counteraction can exquisitely regulate cellular iron levels. Intracellular iron is trafficked throughout the body and transferred to many enzymes by iron carrier proteins, including poly(rC)-RNA-binding protein 1 (PCBP1) or poly(rC)-RNA-binding protein 2 (PCBP2) [21,22,23]. Iron is an essential cofactor for iron-dependent enzymes that require iron–sulfur clusters for proper function, which transfer an electron to targets [24]. The conjugation of iron with proteins generates cellular energy, promotes DNA synthesis and repair, and transmits oxygen to other cells. However, iron can also impair cellular function due to free radical generation by iron redox cycling [25]. Iron-catalyzed reaction products can induce mutations in the active site of an enzyme, causing carcinogenesis [26]. The Fenton reaction is the main source of free radicals in cells. During the Fenton reaction, Fe^2+^ reacts with hydrogen peroxide (H_2_O_2_), producing Fe^3+^, hydroxyl radical (^•^OH), and hydroxyl ion. In turn, ^•^OH is able to initiate lipid peroxidation by abstracting a hydrogen atom from a polyunsaturated fatty acid (PUFA) with bis-allylic hydrogens (–CH=CH-CH_2_-CH=CH_2_–) in the central methylene group to yield their corresponding hydroperoxides [5]. This process culminates in cell death via ferroptosis, a newly defined iron-dependent cell death [5,27]. Thus, the iron balance must be finely regulated at the cellular level.

Cells have an antioxidant system to protect against nucleophiles. Antioxidants eliminate detrimental ROS by functioning as electrophiles. Interestingly, unlike other organs, neurons do not have enough antioxidant proteins, despite their functional importance in life [28]. For example, nuclear factor erythroid 2-related factor 2 (Nrf2), the master regulator of the antioxidant system, is weakly expressed in neurons [29,30]. Although unclear, this defect may result from the development of the neuronal cell. Maintaining an appropriate level of ROS is critical to avoid axonal degeneration due to a high level of oxidative stress (OS) and axonal growth inhibition induced by a low level of OS [31]. Astrocytes provide antioxidant support to neighboring neurons by releasing glutathione (GSH), a potent antioxidant, into the extracellular space [32,33]. Moreover, Nrf2 can play a role in translational regulation as RNA-binding proteins, such as FTH1 [34]. Meanwhile, Nrf2 can inhibit neuroinflammation by suppressing nuclear factor kappa-light-chain-enhancer of activated B cells (NF-κB) activation via hindering the degradation of nuclear factor of kappa light polypeptide gene enhancer in B-cell inhibitor α (IκB-α). IκB-α can prevent NF-κB translocation to the nucleus.

Neuroinflammation is a cellular defensive response against antigens in the central nervous system (CNS), primarily mediated by microglia, astrocytes, endothelial cells, and pericytes. Neuroinflammation enhances the immune system and increases the penetration of endothelial tissues by immune cells. In addition, neuroinflammation reduces antigens’ proliferation. Neuroinflammation is a crucial hallmark of neurodegenerative disease [35]. Cellular ROS or extracellular antigens initiate neuroinflammation. NF-κB promotes inflammatory cytokines, such as IL-6, TNF-α, and IL-1β [36], and forms inflammasomes to maintain normal conditions [37]. However, chronic inflammation induces apoptosis and neurodegenerative diseases, accompanied by increased OS. Increased OS can promote mitochondrial dysfunction and disease progress caused by chronic inflammation [38]. OS can cause aggregated forms of proteins, including amyloid-beta (Aβ), neurofibrillary tangles (NFTs), and alpha-synuclein (α-syn). Especially, 4-hydroxy-2,3-*trans*-nonenal (4-HNE), an end-product of lipid peroxidation, is a key molecule to form detrimental proteins during iron-mediated neuroinflammation. The Fenton reaction facilitates lipid peroxidation and forms 4-HNE as a final product [39]. 4-HNE has reactive bonds and can cause conformational change while producing aggregated forms of Aβ fibril, NFT, or α-syn [40,41]. Inflammation responses can be regulated by antioxidant function (e.g., Nrf2) or post-transcriptional modification (e.g., silent information regulator 2-related enzyme 1, SIRT1).

Considering that studies of ferroptosis have newly elucidated iron’s role in cell death, the present review aims to describe the relationship between intracellular iron homeostasis and neuroinflammation based on recent studies and findings.

## 2. Intracellular Iron Homeostasis

**Figure 1:**
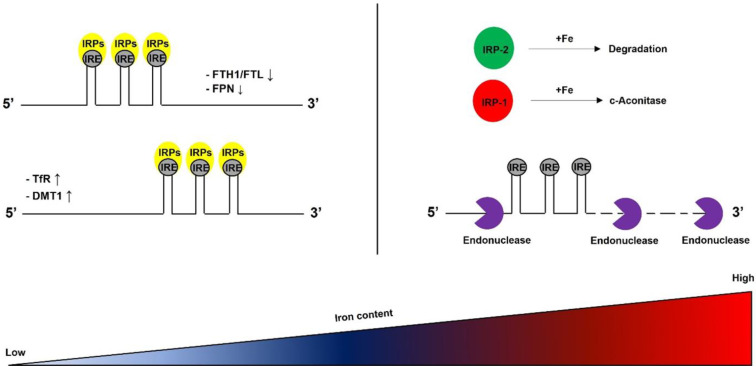
The IRP/IRE system. IRPs consist of two proteins, IRP1 and IRP2. Under iron-rich conditions, iron forms iron–sulfur clusters. Iron–sulfur clusters bind to IRP1. IRP1 acts as c-aconitase. Additionally, iron–sulfur clusters bind to FBXL5 (not described) and mediate IRP2 ubiquitination-dependent degradation. Eventually, inhibition of IRPs leads to the degradation of iron uptake-related mRNAs by the endonuclease. By contrast, under iron shortage conditions, IRPs bind to the IRE within mRNA. This stabilizes the mRNAs or prevents their translation in the nucleus. DMT1, divalent metal transporter 1; FPN1, ferroportin 1; FTH1, ferritin heavy chain; FTL, ferritin light chain; IRE, iron-responsive element; IRP, iron-regulatory protein; IRP1, iron-regulatory protein 1; IRP2, iron-regulatory protein 2; Tfr, transferrin receptor.

The IRP/IRE system regulates intracellular iron homeostasis. IRPs consist of IRP1 and IRP2, possessing RNA-binding capability. IRPs bind to the IRE in the 5′-untranslated region (5′-UTR) or the 3′-untranslated region (3′-UTR) on mRNA and regulate the translation stage [42,43] (Figure 1).

**Figure 2:**
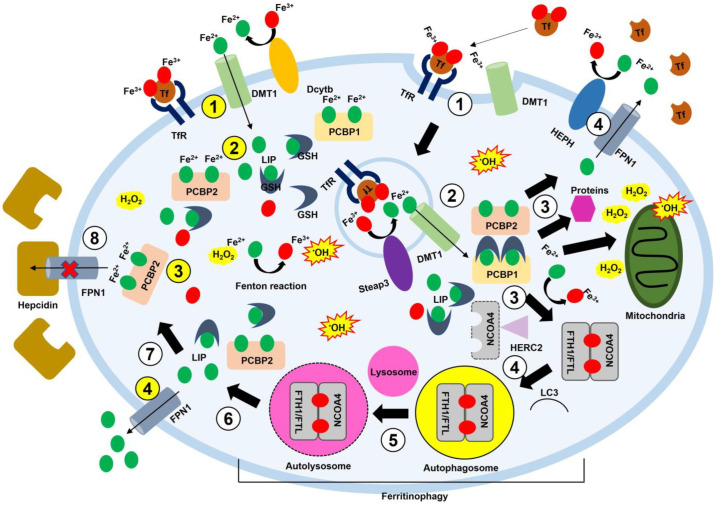
Cellular iron regulation in ferritinophagy. Fe^3+^ is reduced to Fe^2+^ via Dcytb, and Fe^2+^ is then transported into cells via Tf–Tfr or DMT1. Oxidized Fe^3+^ is encapsulated by vesicles called endosomes. Next, Steap3 in the vesicles reduces Fe^3+^ to Fe^2+^ and releases it into the cytoplasm. Fe^2+^ binds to PCBP1 or PCBP2 and is delivered to FTH1, the mitochondria, or FPN1. FTH1 interacts with NCOA4 to store iron. Meanwhile, the interaction between hepcidin and FPN1 blocks the leakage of intracellular iron. When iron is deficient, the FTH1–NCOA4 complex releases iron through ferritinophagy. When iron is repleted, FPN1 exports iron into the extracellular space. In the extracellular space, Fe^2+^ is oxidized to Fe^3+^ by HEPH. Intracellular iron responds to H_2_O_2_ and produces ^•^OH. ROS damages organelles. A white circle with numbers means iron movement by endocytosis. A yellow circle with numbers shows iron movement through a channel. DMT1, divalent metal transporter 1; DcytB, duodenal cytochrome B; Fe^2+^, ferrous iron; Fe^3+^, ferric iron; FTH1, ferritin heavy chain; FTL, ferritin light chain; FPN1, ferroportin 1; GSH, glutathione; HEPH, hephaestin; HERC2, HECT domain and RCC1-like domain 2; ^•^OH, hydroxyl radical; H_2_O_2_, hydrogen peroxide; LC3, microtubule-associated protein 1A/1B-light chain 3; LIP, labile iron pool; NCOA4, nuclear receptor coactivator 4; PCBP1, poly(rC)-binding protein 1; PCBP2, poly(rC)-binding protein 2; ROS, reactive oxygen species; Steap3, six-transmembrane epithelial antigen of prostate family member 3; Tf, transferrin; TfR, transferrin receptor.

During an iron shortage, iron levels are increased by iron influx proteins, such as DMT1, Tf, TfR, and hepcidin. By contrast, iron-efflux-related proteins, such as FPN1, increase under iron-replete conditions. The IRP/IRE system finely regulates these opposed processes. Once iron enters the intracellular space, iron is trafficked by carrier proteins, such as PCBPs, to FTH1/FTL for storage and enzymes for activation. When cellular iron is lacking, FTH1/FTL vesicles release iron to the cytoplasm via NCOA4-mediated ferritinophagy to increase cellular iron contents (Figure 2).

A recent study showed that PCBP1 knockdown could promote ferritiniophagy and lipid peroxidation via binding to the 3′-UTR on beclin 1 (BECN1) mRNA and arachidonate 15-lipoxygenase (ALOX15) mRNA [44]. Although the process of intracellular iron homeostasis and related molecules are known, and new functions of the molecules have been discovered, more studies are needed about the interplay between iron redox homeostasis and neuroinflammation. Thus, this section describes the interaction between iron-related molecules and inflammation.

### 2.1. Hepcidin

Hepcidin is a peptide hormone produced by the liver in response to increased iron levels and inflammation. Hepcidin is involved in iron homeostasis, absorbing dietary iron, releasing recycled hemoglobin iron from macrophages, and transferring stored iron from hepatic cells [45,46]. Inflammation induces hepcidin release and reduces blood iron (i.e., hypoferremia). This increases host resistance to microbial infection and results in anemia. Hepcidin controls cellular iron efflux by interacting with FPN1. The hepcidin–FPN1 response promotes iron uptake [47] (Figure 2). The transcription of hepcidin is mainly regulated by the bone morphogenetic protein (BMP)/suppressor of mothers against the decapentaplegic (SMAD) pathway [48]. A high iron level stimulates BMP6 expression and leads to hepcidin expression by binding to a BMP-responsive element on the hepcidin gene promoter. An increase in hepcidin hinders iron efflux from the cell. Hepcidin levels are closely linked to IL-6 levels. IL-6 increases hepcidin and accumulates iron in the intracellular space while promoting the degradation of FPN1 by hepcidin [49,50]. Accumulated iron in the cell increases the Fenton reaction and ultimately produces excessive ROS, causing inflammation and cellular damage [51,52].

### 2.2. NCOA4

NCOA4 is a selective cargo receptor in ferritinophagy. NCOA4 finely regulates cellular iron homeostasis by anticipating the autophagic degradation of ferritin. Under iron-replete cellular conditions, HERC2-mediated ubiquitylation facilitates the turnover of NCOA4. However, under iron-deficient cellular conditions, NCOA4 is stabilized, thereby promoting ferritinophagy, a type of autophagy, by forming an autophagosome and directing it to the lysosome, which, in turn, increases cellular iron levels [20]. Thus, two selective processes occur according to whether NCOA4 binds to iron. In cells with excess iron, the direct binding of cytosolic iron to NCOA4 mediates its interaction with HERC2 and subsequent degradation, and ferritin is not degraded, thus retaining its stored iron. NCOA4-mediated iron homeostasis also facilitates ferroptosis by increasing cellular iron levels via ferritinophagy [19,53] (Figure 2).

### 2.3. PCBPs

PCBPs are multifunctional proteins that regulate gene expression and bind to iron to form delivery complexes [54]. These complexes deliver iron to other molecules requiring iron for activation. PCBP1 and PCBP2 are essential to maintain the LIP in cells. PCBP2 interacts with DMT1 and FPN1 and directly regulates Fe^2+^ trafficking in and out of the cytosol [55] (Figure 2), whereas PCBP1 plays various roles in the regulation of gene expression as a major iron chaperon [22,44,55,56]. A recent study showed that PCBP1 could regulate ferritinophagy via the interaction between BECN1, an autophagy regulator protein, and PCBP1. PCBP inhibited BECN1 translation by binding to the CU-rich elements in the 3′-UTR of BECN1 mRNA. This binding hampered microtubule-associated protein 1A/1B-light chain 3 (LC3) from forming autophagosomes [44]. In addition, inhibiting PCBPs leads to an iron shortage response because PCBPs cannot deliver iron to iron-related proteins using iron as a cofactor. Although the extracellular iron continuously enters cells, BECN1 promotes the formation of autophagosomes to release stored iron due to the absence of iron delivery proteins interacting with LC3 and NCOA4. In the last stage, autophagosomes fuse lysosomes, called autolysosomes, and release iron into the cytoplasm [19]. Increased iron can expedite the Fenton reaction, and increased ROS damages mitochondria. This aggravates an iron famine because mitochondria can induce the iron starvation response [57,58,59]. Moreover, constitutive deletion of PCBP1 and PCBP2 genes results in early embryonic lethality in mice [60]. Especially, PCBP1 can form a PCBP1–GSH–Fe^2+^ complex and balance the level of cytosolic LIP while delivering Fe^2+^ to an enzyme or ferritin. This process decreases the production of cellular ROS by the Fenton reaction [61,62] and ultimately attenuates lipid peroxidation via NRf2 activation.

### 2.4. IRP/IRE System

The IRP/IRE system consists of IRP1, IRP2, and IRE. IRP1 and IRP2 are the core molecules responsible for iron homeostasis. IRP1 and IRP2 bind to the specific region of target mRNAs called IREs [42]. Under iron deficiency conditions, IRP1 and IRP2 bind to IREs in the UTRs of the iron homeostasis-related mRNAs: ferritin, FPN1, and TfR. The binding of IRPs to the 5′-UTR of IREs in ferritin and FPN1 blocks translation initiation by interfering with the recruitment of the small ribosomal subunit [43]. In contrast, IRPs work differently with TfR mRNA. IRPs protect TfR mRNA from nucleolytic degradation by binding to its 3′-UTR. These reciprocal effects boost iron uptake and repress iron efflux. Under iron-replete conditions, the lack of interaction between IRPs and IREs increases the synthesis of ferritin and FPN1. However, it does not decrease TfR synthesis because TfR mRNA is degraded by endonuclease [63] (Figure 1). As a result, the iron uptake decreases, but the export of iron increases. Meanwhile, activation of the IRP/IRE system can be diminished by ROS. This results in iron deficiency in cells.

### 2.5. DMT1

DMT1 (SLC11A2) transports Fe^2+^ out of endosomes. Ferrireductases on the cell surface reduce most non-Tf-bound iron and then enter the cytosol by DMT1. Expression of DMT1 is elaborately managed in an iron-dependent manner. DMT1 mRNA has the IRE region in the 3′-UTR, and IRPs bind to IREs under iron deficiency [64]. The binding of IRPs to IREs stabilizes DMT1 mRNA and increases DMTI1 synthesis. There is also the non-IRE-containing region on DMT1 mRNA. Alternative splicing determines DMT1 fates, such as DMT1-I with IRE or DMT1-II without the IRE. The DMT1-II isoform is unresponsive to post-transcriptional regulation by intracellular iron concentration because it does not include the IRE [65,66]. Most cells implement the Tf–TfR-mediated process to uptake iron. The Tf–TfR complex forms an endosome with DMT1 and six-transmembrane epithelial antigen of prostate family member 3 (Steap3), acidified to pH 5.5–6.0 via an ATP-dependent proton pump [67]. The Tf–Fe^3+^ complex is released from Tf due to low pH, and then Steap3 reduces Fe^3+^ to Fe^2+^, transferring Fe^2+^ into the cytosol using DMT1 [68]. This process provides cells with Fe^2+^ associated with iron delivery proteins, such as PCBP1 and PCBP2, in the cytosol (Figure 2). DMT1 contributes to the pathogenesis of Parkinson’s disease (PD). Julio et al. suggested that DMT1 expression was increased in PD model mice and patients with PD. In contrast, mutated DMT1 protected rodents from parkinsonism induced by treatment with 1-methyl-4-phenyl-1,2,3,6-tetrahydropyridine (MPTP) and 6-hydroxydopamine [69]. Given that inflammatory cytokines (e.g., TNF-α and IFN-γ) increased DMT1 expression [70], it is reasonable for DMT1 to correlate with inflammation associated with PD development. A study showed that glial cells, activated by inflammatory cytokines, promoted PD progress [71]. Pioglitazone (a peroxisome proliferator-activated receptor alpha [PPAR-α] agonist) effectively attenuated the loss of dopaminergic neurons in substantia nigra in mice by suppressing MPTP-induced microglial activation. Interestingly, caspase inhibitors could not inhibit the degenerative process when dopaminergic neurons were already engaged in apoptosis or autophagic degeneration [72]. Instead, it was efficient for dopaminergic neurons, yet arrived at the final stage [73]. This means that inhibition of DMT1-induced inflammation may impact cell stress during PD, and therapy mainly focuses on the preventive aspect by regulating inflammation.

### 2.6. Ferritin

**Figure 3:**
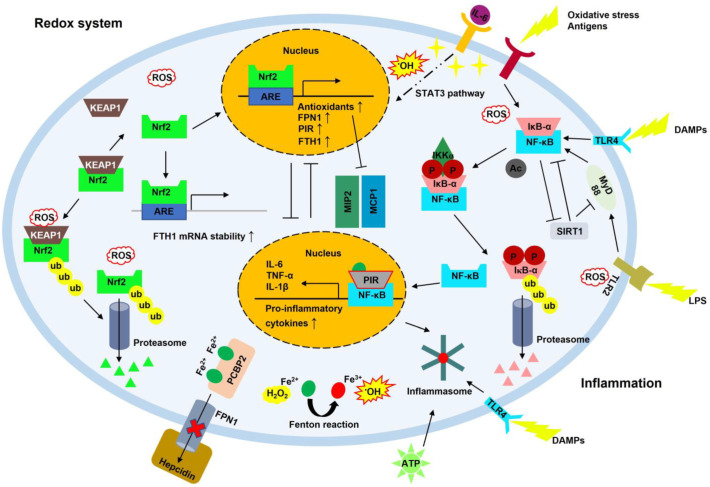
The regulation of cellular redox balance and inflammation. In redox regulation, ROS produced by IL-6 or the Fenton reaction promotes the dissociation of Nrf2 from Keap1 and activates Nrf2. Activated Nrf2 is translocated to the nucleus and initiates the transcription of antioxidant enzymes and proteins requiring iron. This process protects cells from ROS. During inflammation, ROS, DAMPs, or LPS activate NF-κB signal transduction by eliminating IκB-α via ubiquitination. NF-κB moves to the nucleus and induces the transcription of pro-inflammatory cytokines. In this process, inflammasomes are activated, and inflammation is increased. To prevent excessive inflammation, the Nrf2 pathway is activated, which suppresses inflammation-related proteins, such as inflammasomes, MIP2, MCP1, and the NF-κB pathway. Additionally, SIRT1 acts as a regulator and inhibits the activation of NF-κB. NF-κB also regulates the activation of the uncontrolled redox system by inhibiting Nrf2 activation. ARE, antioxidant response element; ATP, adenosine triphosphate; DAMP, damage-associated molecular pattern; FPN1, ferroportin 1; FTH1, ferritin heavy chain; ^•^OH, hydroxyl radical; H_2_O_2_, hydrogen peroxide; IκB-α, nuclear factor of kappa light polypeptide gene enhancer in B-cells inhibitor, alpha; IKKα, IκB kinase alpha; IL-1β, interleukin-1β; IL-6, interleukin-6; KEAP1, Kelch-like ECH-associated protein 1; LPS, lipopolysaccharide; MCP1, monocyte chemoattractant protein 1; MIP2, macrophage inflammatory protein 2; MyD88, myeloid differentiation primary response protein 88; NF-κB, nuclear factor-kappa B; Nrf2, nuclear factor erythroid 2-related factor 2; P, phosphorylation; PCBP2, poly(rC)-binding protein 2; PIR, pirin; ROS, reactive oxygen species; SIRT1, silent information regulator factor 2-related enzyme 1; STAT3, signal transducer and activator of transcription 3; TLR2, Toll-like receptor 2; TLR4, Toll-like receptor 4; TNF-α, tumor necrosis factor-alpha; Ub, ubiquitin.

Ferritin is the main iron storage protein consisting of 24 subunit shells. It has two distinct subunits with different amino acid sequences, designated as FTH1 and FTL. Ferritin synthesis is regulated at the post-translational level through the IRP/IRE system, α-syn, and amyloid precursor protein (APP) [74]. The efficiency of IRE binding to ferritin mRNA is determined by iron (IRP1) and the redox status (IRP2). When iron levels are high, IRP1 forms an iron–sulfur cluster and activates aconitase. However, IRP1 loses RNA-binding activity [75]. IRP2 does not have an iron–sulfur cluster and is regulated by the ubiquitin–proteosome system (UPS) by an E3 ubiquitin ligase complex [76] (Figure 1). Heme is also known to regulate ferritin synthesis. This occurs via BTB and CNC homology 1 (Bach1) binding and IRP2 [77]. FTH1 has a di-iron ferroxidase center that oxidizes Fe^2+^ to Fe^3+^, whereas FTL is considered to form the nucleation site in the mineral iron core [16]. The ferritin complex (FTH1 and FTL) can contain a few hundred to five thousand iron atoms [78]. Fe^2+^ is oxidized to Fe^3+^ via the ferroxidase in FTH1, and subsequently, Fe^3+^ moves toward the nucleation site in FTL and is mineralized and stored. This process is important for efficiency because iron mineralization of ferritin (specifically, FTL) can foster iron oxidation and accelerate circulation between Fe^2+^ and Fe^3+^ in the ferritin complex. However, FTL cannot oxidize Fe^2+^ to Fe^3+^ [79,80] (Figure 2). Under iron starvation conditions, the ferritin complex releases stored iron by promoting autophagy (i.e., ferritinophagy) [19,53]. Increased iron levels help to maintain cellular iron levels and activate iron-dependent enzymes, but excessive iron can increase ROS generation through the Fenton reaction and ultimately induce cell death due to failure in redox control (i.e., ferroptosis) [5,81]. During the inflammation process, ferritin synthesis is indirectly promoted by the IL-6–signal transducer and activator of transcription 3 (STAT3) pathway via hepcidin [82,83] (Figure 3).

IL-1β, IL-6, and TNF-α induce ferritin synthesis by increasing hepcidin transcription [83,84]. Increased ferritin synthesis often leads to hyperferritinemia in serum [84]. The role of extracellular ferritin is still unclear, but several theories are suggested: an iron carrier [85,86,87], to promote angiogenesis [88], to regulate the immune response and inflammatory signaling [82,89,90,91,92,93,94,95,96]. In other words, ferritin helps to decrease stress originating from iron and to maintain a normal immune system during inflammation.

### 2.7. Ferroportin

**Figure 4:**
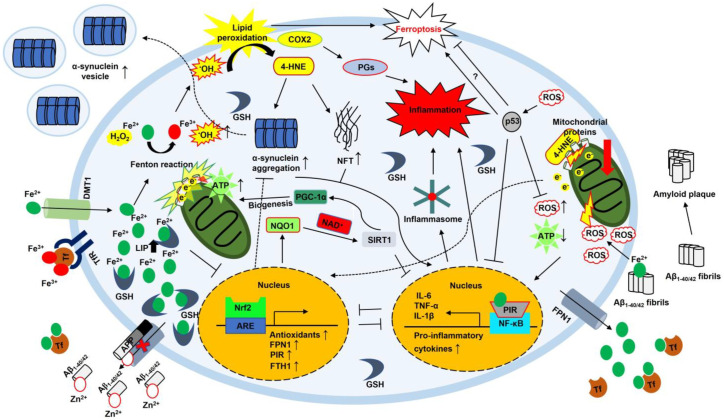
The interplay between iron homeostasis and inflammation in AD and PD. Increased cellular iron accelerates ^•^OH production via the Fenton reaction. Excessive ^•^OH increases lipid peroxidation, producing 4-HNE and activating COX2. 4-HNE promotes α-syn aggregation and continuously generates NFTs. These products are released to the extracellular space with the vesicles or activate the inflammatory response in cells. Additionally, 4-HNE can induce mitochondrial dysfunction by conjugating with mitochondrial proteins, causing electron leakage and enhancing ROS production. Ultimately, this response leads to the activation of the Nrf2-mediated antioxidant response. ROS can stimulate p53. p53 inhibits the NK-κB pathway and reduces ROS. However, there is still controversy about whether p53 prevents ferroptosis. COX2 is a pro-inflammatory enzyme. In addition, iron-binding to Aβ_1-40/42_ fibrils drastically promotes the production of ROS. This damages the mitochondria and boosts ROS production. Ultimately, this cascade leads to the NF-κB-mediated inflammatory response and inflammasome formation. Meanwhile, NQO1 facilitates SIRT1 activation by providing more NAD^+^. SIRT1 promotes mitochondrial biogenesis by activating PGC1-α. Interaction between Aβ_1-40/42_ and Zn^2+^ increases cellular iron content by blocking FPN. α-syn, alpha-synuclein; Aβ, amyloid β; AD, Alzheimer’s disease; APP, amyloid precursor protein; ARE, antioxidant response element; ATP, adenosine triphosphate; COX2, cyclooxygenase 2; DMT1, divalent metal transporter 1; e^−^, electron; Fe^2+^, ferrous iron; Fe^3+^, ferric iron; FPN1, ferroportin 1; FTH1, ferritin heavy chain; 4-HNE, hydroxy-2,3-*trans*-nonenal; H_2_O_2_, hydrogen peroxide; ^•^OH, hydroxyl radical; IL-1β, interleukin-1β; IL-6, interleukin-6; LIP, labile iron pool; NAD^+^, nicotinamide adenine dinucleotide; NQO1, NAD(P)H quinone dehydrogenase 1; NF-κB, nuclear factor-κB; NFT, neurofibrillary tangle; Nrf2, nuclear factor erythroid 2-related factor 2; p53, tumor protein P53; PD, Parkinson’s disease; PGC-1α, peroxisome proliferator-activated receptor gamma coactivator 1-alpha; PIR, pirin; PGs, prostaglandins; ROS, reactive oxygen species; SIRT1, silent information regulator factor 2-related enzyme 1; Tf, transferrin; Tfr, transferrin receptor; TNF-α, tumor necrosis factor-alpha; Zn^2+^, zinc ion.

FPN1 is the sole iron export protein. When iron is overloaded, FPN1 promotes iron efflux. Fe^2+^ binds to the PCBP2 protein and is then transported to FPN1. This balances cellular iron levels [47,97,98]. The degradation of FPN1 is closely related to hepcidin, as mentioned above [47,49]. A lack of FPN1 increases the amounts of intracellular iron and facilitates the Fenton reaction [99] (Figure 2). ROS generated by the Fenton reaction attack PUFAs and promote lipid peroxidation by producing lipid peroxyl radicals. Eventually, lipid peroxyl radicals lead to ferroptosis. Accordingly, the expression of FPN1 is tightly regulated in cells [100] (Figure 4).

### 2.8. Neuroinflammation

Neuroinflammation in the CNS depends on specific cell types: microglia, astrocytes, endothelial cells, and pericytes. Additionally, disruption of the blood–brain barrier leads to the inflammatory response via macrophages [101]. Iron accumulation is identified in many neurodegenerative diseases, including Alzheimer’s disease (AD), PD, and amyotrophic lateral sclerosis (ALS). In these neurodegenerative diseases, inflammation is promoted in glial cells and neurons, but there is still a lack of understanding of the role of iron in neuroinflammation. Considering the active redox trait of iron, increased iron levels in the intracellular space can have detrimental effects because they can produce ^•^OH through the Fenton reaction and subsequently damage biomolecules, causing cell death [102]. In ferroptosis, ^•^OH induces lipid peroxidation and promotes inflammation by activating cyclooxygenase-2 (COX2) [103,104]. Recently, researchers have been studying the relationship between ferroptosis and neurodegenerative diseases [105,106]. However, a few studies have shown a relationship between iron and neuroinflammation.

### 2.9. NF-κB

NF-κB consists of five transcription factors; NF-κB1 (p105/p50), NF-κB2 (p100/p52), RelA (p65), RelB, and c-Rel. Activated NF-κB participates in the inflammatory response by promoting pro-inflammatory genes. Activation of NF-κB leads to two distinct pathways: canonical and noncanonical. These two distinct pathways have different stimuli. In the canonical pathway, inflammatory stimuli, such as cytokines, antigens, and damage-associated molecular patterns (DAMPs), release p65/p50 dimers from IκBα, phosphorylating IκBα and degrading it through the UPS. Free p65/p50 dimers are translocated to the nucleus, activating the transcription of NF-κB target genes [107]. The trigger is a subset of tumor necrosis factor receptor (TNFR) superfamily members in the noncanonical pathway. They activate NF-κB-inducing kinase (NIK), and NIK phosphorylates IκB kinase alpha (IKKα). Following the phosphorylation cascade, p52/RelB enters the nucleus and promotes the expression of NF-κB target genes. NF-κB signaling is important for immune cell development [108] (Figure 3). Given that Toll-like receptors (TLRs, an inducer of inflammatory response) of microglia are highly expressed in AD [109], it is reasonable for NF-κB to be involved in AD progression. TLRs promote the canonical NF-κB signal transduction, which leads to chronic inflammation in AD due to stimuli, such as cytokines and Aβ plaques [110]. Patients with PD showed increased levels of OS. Immunohistochemical analyses of brain sections with PD showed increased activation of NF-κB, consistent with elevated levels of OS and decreased Nrf2 activation [111]. Interestingly, Fe^2+^ is related to excessive abnormal ROS generation in neuroblastoma. Fe^2+^ inhibits the Nrf2 signal pathway, exacerbates mitochondrial dysfunction, and promotes α-syn aggregation [112] (Figure 4). Recent studies revealed that severe OS could promote α-syn proteostasis [41,113], indicating that OS increased by Fe^2+^-induced inhibition of Nrf2 may promote neuroinflammation by interfering with the Nrf2 countereffect on NF-κB activation in PD. In contrast, NF-κB is also known to induce FTH1 expression. Increased FTH1 can indirectly inhibit ROS accumulation by sequestrating iron and reducing the Fenton reaction, leading to the attenuation of apoptosis [114]. This process can oppose the detrimental role. The final effect of these two opposing roles may be determined by the antioxidant level.

### 2.10. SIRT1

Sirtuins are class III (NAD^+^-dependent) histone deacetylases. In mammals, the sirtuin family is comprised of seven members, SIRT1–SIRT7 [115,116]. The sirtuins regulate diverse genes through epigenetic modification. This regulation mainly involves genomic stabilization, stress response, apoptosis, metabolism, senescence, proliferation, and inflammation [117,118,119]. Especially, SIRT1 is well studied because of its various physiological functions. SIRT1 promotes the epithelial–mesenchymal transition (EMT) process in cancer while endowing more aggressive traits to cancer but decreasing the antioxidant system [120,121,122,123]. SIRT1 promotes neuronal fortification during neuroinflammation and neurodegenerative diseases [124,125]. Once SIRT1 is activated, for example, by using NAD^+^ produced by the enzymatic action of NAD(P)H quinone dehydrogenase 1 (NQO1), it can inhibit NF-κB by deacetylating the p65 subunit of NF-κB and vice versa (Figure 3). Antagonistic crosstalk between SIRT1 and NF-κB is finely regulated to maintain cellular homeostasis [126]. Moreover, several studies showed that SIRT1 weakened neuroinflammation by inhibiting the TLR pathway. Resveratrol, a SIRT1 activator, decreased neuroinflammatory cytokines, such as IL1β and TNF-α, and improved spatial reference memory through repression of TLR2–myeloid differentiation primary response protein 88 (MyD88)–NF-κB signal transduction [127] (Figure 3). Recent evidence demonstrated the protective effects of SIRT on inflammation in AD and PD [128,129]. In AD, resveratrol decreases the expression of Aβ, promotes deacetylation of the tau protein, and represses apoptosis [130,131,132,133,134,135]. Overexpression of SIRT1 in the hippocampus enhanced learning and memory by reducing Aβ and tau in the triple-transgenic (3xTg) AD mouse model [136]. However, considering that resveratrol is not a SIRT1-specific activator, further study is needed to show the effects of SIRT1 on AD using a SIRT1-specific activator, SRT1720 [120]. In PD, resveratrol decreases apoptosis by inhibiting NF-κB and degrading α-syn via deacetylation of LC3 [137,138,139,140]. Additionally, a recent study showed that SIRT1 could promote mitochondrial biogenesis by activating peroxisome proliferator-activated receptor-gamma coactivator 1 (PGC-1) [141] (Figure 4).

### 2.11. Inflammasome

Inflammasomes are cytosolic molecular complexes that promote inflammatory responses to activate immune defenses. Inflammasomes are classified as nucleotide-binding oligomerization-like receptor (NLR) domain and leucine-rich repeat and pyrin domain-containing protein 1 (NLRP1), NLRP3, NLR family CARD domain-containing 4 (NLRC4), AIM2, and pyrin inflammasomes [142]. Inflammasomes consist of the NLR protein or AIM2-like receptor, apoptosis-associated speck-like protein containing a CARD (ASC), and pro-caspase-1. The NLR protein can sense an intracellular signal that promotes the formation of inflammasomes. Once inflammasomes are formed, activated caspase-1 mediates the catalytic cleavage and release of the pro-forms of pro-inflammatory cytokines, such as IL-1β and IL-18 [143]. In the CNS, inflammasome formation occurs in microglia, neurons, and astrocytes. Especially, NLRP3 inflammasome plays a crucial role in the neuroinflammation response [144]. NLRP3 inflammasome and NLRP3-dependent inflammatory cytokines are found in the periphery plasma of patients with PD [145]. Aggregated α-syn released from neurons can interact with TLRs in microglia, which activates NLRP3 inflammasome in microglia. In turn, NF-κB is translocated to the nucleus, leading to an increase in pro-inflammatory cytokines. Furthermore, pathological α-syn impairs mitochondrial homeostasis, interfering with protein transport via the translocase of the outer membrane (TOM) receptor, such as TOM20, and inhibiting SIRT3 activation in the mitochondria of microglia [146]. Meanwhile, mitochondrial ROS activates nicotinamide adenine dinucleotide phosphate oxidase 2 (NOX2) in microglia, resulting in microglial activation and neurotoxicity [147], ultimately leading to neuroinflammation and neuronal dysfunction [148,149,150]. However, another study reported that macrophages could regulate the inflammatory response via the NF-κB–p62-mitophagy pathway (a type of autophagy). NF-κB promotes p62 activation, an adaptor that binds polyubiquitinated proteins and helps to form autophagosomes [37]. Mitophagy eliminates damaged mitochondria, restrains NLRP3 activation, and, ultimately, attenuates the inflammatory response [151]. In AD, there are two main inflammasome activation pathways: the MYD88-dependent pathway (signal 1) and the ATP-dependent pathway (signal 2). The MYD88-dependent pathway utilizes DAMPs as a trigger. DAMPs stimulate NF-κB activation via TLRs in microglia (Figure 3). This increases the production of pro-inflammatory cytokines and facilitates the formation of inflammasomes. Activated inflammasomes trim pro-inflammatory cytokines into active forms. IL-1β is intimately linked to the pathogenesis of AD. Among other pro-inflammatory cytokines, IL-1β levels are increased in patients with AD. In signal 2, P2X purinergic receptor 7 (P2X7R), a trimeric ATP-gated cation channel, is a protagonist in forming inflammasomes. A study reported that P2X7R is related to chronic inflammatory neurological disorders [152]. P2X7R was highly expressed in immune cells, such as macrophages, mast cells, microglia, and oligodendrocytes, but to a lesser extent in astrocytes and neurons. In high-ATP conditions, P2X7R was activated, promoting the activation of inflammasomes [153].

### 2.12. NRF2

Nrf2 is known as a master regulator of cytoprotection against oxidative and xenobiotic stresses [154]. Nrf2 is a ubiquitously expressed redox-sensitive transcription factor with an important role in redox homeostasis and cell inflammation. Nrf2 promotes the expression of antioxidant enzymes and anti-inflammatory molecules [155,156,157]. Under normal conditions, Nrf2 is maintained at low basal levels in the cytoplasm because of its degradation by the UPS. In a normal state, Kelch-like ECH-associated protein 1 (Keap1), an adaptor protein for a cullin 3 (Cul3)-based ubiquitin E3 ligase, tightly binds to Nrf2, targeting Nrf2 for degradation by the proteasome [158,159,160]. However, OS and Nrf2-inducing chemicals reduce the E3 ligase activity of the Keap1–Cul3 complex and liberate Nrf2 from the Nrf2–Keap1 complex. This stabilizes Nrf2 against degradation, and Nrf2 is translocated to the nucleus. Continuously, Nrf2 binds to the antioxidant response element (ARE) that has the promoter for transcription of phase II detoxifying antioxidant enzymes. Once Nrf2 binds to the ARE motif, antioxidant enzymes are transcribed, and cellular antioxidant systems are simultaneously activated to protect cells from harmful molecules [161,162] (Figure 3). Activation of antioxidants is intertwined with inflammation. They block inflammatory mediators, including IL-6, TNF-α, monocyte chemoattractant protein 1 (MCP1), and macrophage inflammatory protein 2 (MIP2) [163]. This process is important in the progression of neurodegenerative diseases. A study showed that inflammatory markers, such as inducible nitric oxide synthase (iNOS), TNF-α, and IL-6, were increased in the hippocampus of Nrf2-knockout mice [164]. Despite its anti-inflammatory role, Nrf2 has Janus-like roles. On the one hand, Nrf2 inhibits NLRP3 inflammasome by increasing the expression of NQO1, one of the antioxidant enzymes induced by Nrf2, in macrophages [165,166]. On the other hand, Nrf2 has been shown to activate NLRP3 and AIM2 inflammasomes [167]. However, many studies demonstrated that Nrf2 negatively regulated NF-κB and vice versa. Nrf2 negatively influenced NF-κB-induced inflammation in three aspects: degradation of IKKβ by Keap1 [168], inhibition of OS by activation of Nrf2 induced by the cyclopentenone prostaglandin 15d-PGJ2 [169], and forming a complex with the competitive Nrf2 transcriptional coactivator CREB-binding protein (CBP) [170,171]. The result of three aspects ends in the inactivation of NF-κB. Furthermore, Nrf2-induced heme oxygenase 1 (HO-1) prohibited the translocation of NF-κB to the nucleus [172]. The disease phase affects the Nrf2 response. In the frontal cortex of patients with AD, NQO1 activity was increased during the initial stages of AD but reduced or maintained in the latter stage of AD [173]. This inducible cellular defense system helps cells resist unfavorable environments. In PD, Nrf2 can effectively reduce α-syn aggregation [174], whereas Nrf2 deficiency leads to increased α-syn aggregation, loss of neurons, and enhanced inflammation [175] (Figure 4).

Nrf2 is also closely associated with iron metabolism [176,177,178]. Nrf2 coordinates iron homeostasis within LIPs. Especially, Nrf2 promotes ferritin expression. Nrf2-deficient mice showed lower basal FTH1 and FTL levels than wild-type mice [179,180]. The regulation mechanism was uncovered by Pietsch et al. They proved that Nrf2 is directly bound to the ARE on FTH1 mRNA [181], suggesting that Nrf2 activation promotes iron storage and reduces labile iron levels by boosting ferritin expression. Meanwhile, Nrf2 is also involved in FPN1 expression. Nrf2 activation may displace Bach1 and inhibit the transcription of HO-1 and FPN1 genes through direct DNA binding [182]. Other studies suggested that Nrf2 activators (e.g., diethyl malate, sulforaphane) could increase FPN1 mRNA in murine macrophages in an iron-independent manner. Interaction between Nrf2 and FPN1 helped macrophages to offset the suppression of FPN1 mRNA expression following lipopolysaccharide (LPS) treatment [183]. Furthermore, Nrf2 increases pirin (PIR) transcription. PIR is known to regulate NF-κB transcriptional signaling and has an enzymatic redox function. Activation of PIR requires iron as a cofactor to form a PIR–iron complex. The PIR–iron complex alters the allosteric capability of NF-κB to bind to DNA [184,185]. Ultimately, the PIR–iron–NF-κB complex increases the NF-κB transcription of target genes (Figure 4). Nrf2 knockdown in HeLa cells reduced PIR expression, whereas Nfr2 overexpression increased the PIR mRNA level by 30% compared to the control [186]. Overall, Nrf2 activation plays a key role in cellular iron homeostasis and helps protect cells from oxidative damage.

## 3. Conclusions and Perspectives

Iron homeostasis is critical for the functioning of cells and organisms. Impairment of iron homeostasis can have devastating effects on human health. Ferroptosis induced by an imbalanced iron level emphasizes the importance of iron homeostasis. ROS generated by the Fenton reaction stimulate cellular antioxidant systems. However, cell damage occurs when the ROS burden exceeds the capacity of the antioxidant systems. Increased IL-6 in the immune response promotes the interaction between hepcidin and FPN1. This response inhibits the utilization of iron, an essential element of antigens. However, this process accelerates detrimental effects by promoting iron uptake instead of enhancing the immune system in extracellular space. Cellular iron shortage can also facilitate iron uptake through the DMT1–Tf–TfR complex and stimulates ferritinophagy via NCOA4. Increased intracellular iron is transferred to iron-dependent enzymes and inhibits ferritin (FTH1/FTL) turnover through PCBPs. Nevertheless, excessive iron can accelerate the Fenton reaction and lead to excessive ROS generation, boosting inflammation and cellular damage. Cells initiate the transcription of antioxidants using the Nrf2–ARE pathway to hinder severe injury. In this respect, the IRP/IRE system has a crucial role in the relationship between iron homeostasis and inflammation. Activation of Nrf2 inhibits the NF-κB pathway by preventing the degradation of IκB-α. This hinders the translocation of NF-κB to the nucleus and the transcription of pro-inflammatory cytokines. Prolonged activation of NF-κB promotes chronic inflammation and OS. In AD, Aβ_1–40/42_ binds to redox-active metal ions (Cu^2+^, Zn^2+^, and Fe^2+^) to form Aβ oligomers and, ultimately, Aβ fibrils (components of amyloid plaques). In forming Aβ–metal ions complex, OS and APP increase the cellular iron influx. Interestingly, AD progression is related to ferroptosis. In ferroptosis, iron promotes iron-based lipid peroxidation and ultimately produces 4-HNE. Continuously, 4-HNE induces tau protein aggregation, producing NFTs through modifying tau conformation. Moreover, 4-HNE can conjugate with mitochondrial proteins involved in energy production. This conjugation results in a conformational change and increases electron leakage from the electron transport chain, causing ROS generation. Consequently, this decreases ATP production and increases the level of OS due to mitochondrial dysfunction. In addition, COX2 is activated during ferroptosis and promotes inflammation. In the initial stage of PD, 4-HNE promotes α-syn aggregation. Suppression of the Nrf2 pathway by Fe^2+^ may promote OS and α-syn aggregation due to increased OS in PD. Iron-associated ROS production also facilitates inflammasome formation via NF-κB or P2X purinoceptor 7 (P2XR7) activation. Considering the importance of the antioxidant system, NAD(P)H-dependent enzymes may also be involved in regulating iron-induced inflammation. Enzymes requiring NAD(P)H possess antioxidant properties and a role as an energy provider. As an energy provider, a representative enzyme is NQO1. NQO1 increases NAD^+^ and activates SIRT1. Activated SIRT1 can inhibit NF-κB via deacetylation of p65. This process may decrease OS and inflammation. In addition, PGC-1 activation by SIRT1 may compensate for the loss of mitochondria by promoting mitochondrial biogenesis. This may offer a practical benefit for patients with mitochondria dysfunction.

The relationship between iron and cell death has been known for over 30 years, but advanced research on the mechanism of iron-dependent cell death has recently been achieved in the cancer field. New findings will help to understand iron and diseases. Thus, the interplay between iron, cell death, and inflammation in neurobiology needs to be re-examined considering recent findings. The imbalance of iron homeostasis and excessive inflammation can cause detrimental effects on cells, highlighting the importance of their regulation. Many studies mainly focus on inflammation or the relationship between iron homeostasis and OS because iron-dependent cell death has actively been studied. Iron homeostasis is intimately associated with inflammation. However, the interaction of each molecule will need further study to understand the exact connection between them. Furthermore, considering that many molecules require energy for activation, further examination of iron homeostasis and inflammation is needed from the viewpoint of energy metabolism. This will improve the understanding of neurodegenerative diseases.

## Abbreviations

α-syn	alpha-synuclein
Aβ	amyloid-beta
AD	Alzheimer’s disease
ALOX15	arachidonate 15-lipoxygenase
ALS	amyotrophic lateral sclerosis
APP	amyloid precursor protein
ARE	antioxidant response element
ASC	apoptosis-associated speck-like protein containing a CARD
ATP	adenosine triphosphate
Bach1	BTB and CNC homology 1
BECN1	beclin 1
BMP	bone morphogenetic protein
CNS	central nervous system
COX2	cyclooxygenase-2
CRB	CREB-binding protein
DAMP	damage-associated molecular pattern
DcytB	duodenal cytochrome B
DMT1	divalent metal transporter 1
e^−^	electron
EMT	epithelial–mesenchymal transition
Fe^2+^	ferrous iron
Fe^3+^	ferric iron
FPN1	ferroportin 1
FTH1	ferritin heavy chain
FTL	ferritin light chain
GSH	glutathione
4-HNE	4-hydroxy-2,3-trans-nonenal
HEPH	hephaestin
HERC2	HECT domain and RCC1-like domain 2
HMOX-1	heme oxygenase
H_2_O_2_	hydrogen peroxide
HO-1	heme oxygenase-1
IκB-α	nuclear factor of kappa light polypeptide gene enhancer in B-cells inhibitor alpha
IKKα	IκB kinase alpha
IL	interleukin
iNOS	inducible nitric oxide synthase
IRE	iron-responsive element
IRP	iron-regulatory protein
KEAP1	Kelch-like ECH-associated protein 1
LC3	microtubule-associated protein 1A/1B-light chain 3
LIP	labile iron pool
LPS	lipopolysaccharide
MCP1	monocyte chemoattractant protein 1
MIP2	macrophage inflammatory protein 2
MPTP	1-methyl-4-phenyl-1,2,3,6-tetrahydropyridine
MyD88	myeloid differentiation primary response protein 88
NAD^+^	nicotinamide adenine dinucleotide
NCOA4	nuclear receptor coactivator 4
NF-κB	nuclear factor kappa-light-chain-enhancer of activated B cells
NFT	neurofibrillary tangle
NIK	NF-κB-inducing kinase
NLRP	nucleotide-binding domain and leucine-rich repeat and pyrin domain-containing protein
NOX2	nicotinamide adenine dinucleotide phosphate oxidase 2
NQO1	NAD(P)H quinone dehydrogenase 1
Nrf2	nuclear factor erythroid 2-related factor 2
•OH	hydroxyl radical
OS	oxidative stress
P	phosphorylation
P2X7R	P2X purinergic receptor 7
p53	tumor protein P53
PCBP	poly(rC)-binding protein
PD	Parkinson’s disease
PGC-1α	peroxisome proliferator-activated receptor gamma coactivator 1-alpha
PIR	pirin
PPAR	peroxisome proliferator-activated receptor
PGs	prostaglandins
PUFA	polyunsaturated fatty acid
ROS	reactive oxygen species
SIRT1	silent information regulator factor 2-related enzyme 1
STAT3	signal transducer and activator of transcription 3
Steap3	six-transmembrane epithelial antigen of prostate family member 3
SMAD	suppressor of mothers against the decapentaplegic
Tf	transferrin
TfR	transferrin receptor
TLR	Toll-like receptor
TNF-α	tumor necrosis factor-α
TNFR	tumor necrosis factor receptor
TOM20	translocase of the outer membrane 20
Ub	ubiquitin
UPS	ubiquitin–proteosome system
UTR	untranslated region
Zn^2+^	zinc ion
